# Is the percentage of hormone receptor positivity in HR+ HER2-metastatic breast cancer patients receiving CDK 4/6 inhibitor with endocrine therapy predictive and prognostic?

**DOI:** 10.3389/fonc.2024.1378563

**Published:** 2024-06-18

**Authors:** Merve Keskinkilic, Huseyin Salih Semiz, Tugba Yavuzsen, Ilhan Oztop

**Affiliations:** ^1^ Department of Hematology and Medical Oncology, Emory Winship Cancer Institute, Atlanta, GA, United States; ^2^ Department of Medical Oncology, Dokuz Eylul University Faculty of Medicine, Izmir, Türkiye; ^3^ Department of Medical Oncology, Institute of Oncology, Dokuz Eylul University, Izmir, Türkiye

**Keywords:** CDK 4/6 inhibitor, hormone receptor positivity, metastatic breast cancer, palbociclib, ribociclib

## Abstract

**Purpose:**

There is no clear information in the literature about the relationship between the efficacy of CDK 4/6i combined with ET and HR positivity. However, we know that the longest overall survival was in the ER-strong positive/PR intermediate or strong positive groups. Therefore, we aimed to investigate CDK4/6i treatments that create positivity in HR.

**Methods:**

Patients with the diagnosis of HR+/HER2- MBC who were treated with CDK 4/6i and HR >10% were retrospectively evaluated. To analyze the role of HR positivity, ER was moderately positive (10-49%) and ER was strongly positive (50-100%); PR was grouped as moderately positive (10-49%) and PR strongly positive (50-100%).

**Results:**

Median follow-up of 150 patients included in the study was 15.2 months (95% CI, 2.1-40.9 months). The highest response in the whole group was obtained in the ER-strong positive/PR moderate or strong positive group, and the ER moderate positive/PR moderate or strong group. This was followed by the ER strong positive/PR negative group, and then the ER moderate positive/PR negative group. Although these advantages were not statistically significant, they were numerically higher (ORR: 83.8% vs. 83.3% vs. 77.4% vs. 62.5%, p=0.488, respectively). The highest survival in the whole group was achieved in the ER strong positive/PR moderate or strongly positive group, followed by the ER moderately positive/PR moderate or strongly positive group, the ER strongly positive/PR negative group followed by the ER moderate positive/PR negative group, respectively(p=0.410). However, these advantages were not statistically significant.

**Conclusion:**

As a result, HR+/HER2- MBC patients receiving CDK 4/6i combined with ET suggest that the percentage of HR positivity may have a predictive and prognostic role.

## Introduction

Breast cancer is the most common type of cancer in women and is an important public health problem worldwide (1). Although significant improvements in survival have been achieved in recent years thanks to the advances in systemic treatments, metastatic disease still remains a serious problem affecting prognosis. The main treatment options for metastatic disease have historically been chemotherapy, targeted therapies, and hormone therapy, but in recent years cyclin-dependent kinase 4/6 inhibitor (CDK 4/6i) and immunotherapy have been added to these (2, 3). Breast cancer is classified into three main subgroups according to hormone receptor (HR) (estrogen receptor (ER) and progesterone receptor (PR)) status and human epidermal growth factor 2 (HER2) status: HR+ group, HER2+ group and triple negative group (4). The HR+/HER2- group constitutes approximately two-thirds of patients with metastatic breast cancer (MBC) (5). In this group, endocrine therapy (ET) constitutes the main framework of treatment (6). Aromatase inhibitors (AI) (Anastrozole, Letrozole, Exemestan, etc.), selective estrogen modulators (Tamoxifen), and selective estrogen degraders (Fulvestrant) are widely used as ET (7–9). However, intrinsic or acquired resistance is also encountered in endocrine treatments (10). In order to overcome this problem, combinations with targeted agents, especially CDK 4/6i, have been sought in recent years.

By inactivating the CDK-D-cyclins (CCND) complex, CDK 4/6i increase retinoblastoma protein (pRb), which negatively affects the E2F transcriptional factor, and ultimately induces tumor cell apoptosis by inhibiting cell cycle progression (11). In the vast majority of phase III trials combining CDK 4/6i and ET (AI (Anastrozole, Letrozole) and selective estrogen degraders (Fulvestrant)), progression free survival (PFS) and overall survival (OS) have been significantly improved in the front line as well as in subsequent lines of therapy. Thus, CDK 4/6i have become the main treatment model in the HR+ HER2- patient group with their unique mechanism of action, consistent survival advantages in phase III studies, and different toxicity characteristics (12–14).

It is known that the estrogen signal, which has a fundamental role in breast cancer, has a significant effect on the Cyclin D1-CDK4/6-RB1 complex. This constitutes the rationale for combination therapy based on inhibition of this interaction by combined with ET and CDK 4/6i (15, 16). In this direction, the first studies were carried out in patients with HR+/HER2- MBC (12–14). In general, those with ER positive staining percentage >1% in the pathology material are defined as endocrine sensitive, but those with 1-9% ER+ are called ERlow positive according to the American Society of Clinical Oncology/College of American Pathologists Guideline (ASCO/CAP). However it is well known that these tumors often gain little benefit from ET (17–20). Therefore, in studies evaluating the combination of ET and CDK 4/6i, patients with an ER+ of 10% and above were included (12–14). In these studies, patients were grouped as ER+/PR+ and ER+/PR- according to their HR+, but the percentage of HR positivity was not further categorized in terms of endocrine sensitivity.

On the other hand, when the meta-analysis of the Early Breast Cancer Trialists’ Collaborative Group (EBCTCG) is evaulated it is observed that ER positivity is categorized and the benefit from ET is highest especially in the group with ER+>50% (18). In different studies, it has been reported that the therapeutic effect and survival are correlated with the rate of ER positivity in breast cancer patients who is receiving ET (19–22). Similarly, there is information regarding the predictive and prognostic role of a high percentage of predictive biomarker positivity in other tumor groups. For example, in the TOGA study, in which the addition of trastuzumab to systemic chemotherapy in metastatic gastric cancer was investigated the most benefit was observed in the group with immunohistochemically (IHC) HER2-positive 3+/*in-situ* hybridization (ISH)+ with proportionately less benefit in the IHC 2+/ISH+ and IHC1+/ISH+ subgroups (23). Similarly, it has been reported that the percentage of ALK-positivity in anaplastic lymphoma kinase (ALK)-positive non-small cell lung cancer (NSCLC) is correlated with response and survival (24).

Therefore, when all these results were evaluated together, we suggested that the percentage of hormone positivity in patients receiving CDK 4/6i together with ET may affect the response and overall outcome.

## Methods

### Patient characteristics

In this study, patients who were followed up and treated in Dokuz Eylul University Faculty of Medicine, Department of Medical Oncology between January 01, 2020 and January 01, 2023, with the diagnosis of HR+ HER-2- MBC, who received ET plus CDK 4/6i, were retrospectively evaluated. Demographic characteristics of the patients, complete blood count, biochemical laboratory parameters, clinicopathological features of the tumor were recorded from the hospital database. Patients were included on the basis of the following criteria: (1) patients with breast cancer based on core needle biopsy before treatment; (2) having diagnosed with HR-positive HER2-negative MBC; (3) patients who is receiving CDK 4/6i with (palbociclib or ribociclib) ET a for at least 2 months, (4) performance status (ECOG-PS) ranging from 0 to 2; (5) having complete medical record and follow-up information; (6) be 18 years or older; (7) to be survived more than 3 months. Patients were excluded on the basis of the following criteria: (1) Patients with synchronous and metachronous tumors; (2) having diagnosed with HR-negative HER2-positive and triple-negative breast cancer.

### Ethics committee approval

This study was performed in line with the principles of the Declaration of Helsinki. Approval was granted by Non-Invasive Research Ethics Committee of Dokuz Eylul University Faculty of Medicine (Date: 09.02.2022/No: 2022/05-09).

### Endocrine therapy + CDK 4/6 inhibitor therapy

Patients who were started on CDK 4/6i combined with ET with the diagnosis of HR+ HER2- MBC were included in the study. Those receiving endocrine therapy were categorized as either AI (anastrozole or letrozole) or selective estrogen degrader (fulvestrant). Those receiving CDK 4/6i therapy were also grouped as those receiving palbociclib or ribociclib. Then, the patients were subcategorized as two groups by receipt of AI plus CDK4/6i or fulvestrant plus CDK4/6i.

### Hormone receptor status

ER and PR analyzes of tumor materials of the patients were performed by IHC, based on the American Society of Clinical Oncology/College of American Pathologists Guideline (ASCO/CAP) (17). The results obtained according to the immune reactivity status in the tumor cell nucleus were categorized as follows for both ER and PR: ERnegative: 0% or <1%, ERlow: 1-9%, ERpositive: 10-100%; PRnegative: 0% or 1%, PRlow: 1-9%, PRpositive: 10-100%. To analyze the role of high hormone receptor positivity in this study, ERpositive (10-100%) and PRpositive (10-100%) groups were also categorized as an ERmoderately positive (10-49%) and ERstrongly positive (50-100%); PR moderately positive (10-49%) and PR strongly positive (50-100%). The status of the aforementioned ER and PR analyzes from primary tumor or metastasis was recorded. ER and PR analyzes of all patients were evaluated and recorded before starting CDK 4/6i combined with ET.

### Response and toxicity assessment

Tumor staging was performed according to “Eighth Edition of American Joint Committee on Cancer (AJCC) and the Union for International Cancer Control (UICC) TNM stage classification” (25). Response assessments were made according to the “Response Evaluation Criteria in Solid Tumors (RECIST) v1.1 guidelines” (26). Toxicity assessments were made according to the National Cancer Institute Common Toxicity Criteria (NCI-CTC) (27).

### Statistical analysis

Demographic characteristics, clinicopathological features, and blood sample results were collected from the hospital database. Since our study was a retrospective, cross-sectional study, the sample size was not calculated. In addition to descriptive statistics, Chi-square and Fisher’s exact tests were used for categorical variables in the evaluation of the data. The effect of ER and PR positivity percentage and clinicopathological features of breast cancer on treatment response and survival were analyzed with Chi-square and Fisher’s Exact tests. Student’s t test, Mann-Whitney U test and Kruskal Wallis test were used to determine the differences between the measured variables according to their suitability. As progression-free survival time (PFS), the time from the start of ET plus CDK 4/6i therapy to the date of progression; The overall survival time (OS) was taken as the time from the start of ET plus CDK 4/6i therapy to death/last follow-up date. Kaplan-Meier method and Log-rank test were used for survival analysis. The suitability of the data for normal distribution was evaluated with the Kolmogorov Smirnov test and it was found that it did not have a normal distribution. Therefore, median values were used when reporting OS and PFS data. Therefore, mean values were used when reporting OS and PFS data. The prognostic and predictive effect of ER and PR positivity percentage was analyzed with univariate and multivariate Cox Regression model. The median follow-up time in the study was calculated using the reverse Kaplan-Meier. IBM SPSS for analysis of all data *(Sciences Statistical Package for the Social, version* 24.0) package program was used. Statistical significance was determined as p<0.05.

## Results

### Patient characteristics

A total of 150 patients with HR+ HER2- MBC who received CDK 4/6i combined with ET were evaluated. The median age of the patients was 55.0 years (26.2-90.2), of which 147 (98%) were female and 3 (2%) were male. Of the 147 female patients, 109 (74.1%) were postmenopausal. The most common site of metastasis was bone (n=119, 79.3%), followed by lymph nodes (n=91, 60.7%) and liver (n=42, 28%). The characteristics of the patients are shown in **Table 1**.

**Table 1:** Sociodemographic and clinicopathologic characteristics of patients.

Characteristics	n (%)
Sex	
Female	147 (98%)
Male	3 (2%)
Comorbidity	
None	75 (50%)
One	47 (31.3%)
Two or more	28 (18.7%)
Menopause Status	
Postmenopausal	109 (74.1%)
Pre/peri menopausal	38 (25.9%)
Histological Subtype	
Invasive ductal carcinoma (IDC)	51 (34.0%)
Invasive carcinoma	38 (25.3%)
Invasive lobular carcinoma (ILC)	24 (16.0%)
Mixed type (IDC+ ILC)	17 (11.3%)
Mucinous	5 (3.3%)
Tubuloalveoler	1 (0.7%)
Unknown	14 (9.4%)
Metastasis Site	
Bone	119 (79.3%)
Lymph node	91 (60.7%)
Liver	42 (28%)
Lung	37 (24.7%)
Brain	5 (3.3%)
Others	32 (21.3%)

### CDK 4/6 inhibitor and endocrine therapy

Seventy five (50.0%) of 150 patients received AI plus CDK 4/6i treatment and 75 (50.0%) received fulvestrant plus CDK 4/6i. Patients receiving AI plus CDK 4/6i receive this treatment as first-line therapy while those receiving fulvestrant + CDK 4/6i were being treated in second-line therapy after progression on AI treatment. Median duration of treatment for AI plus CDK 4/6i was 14.0 months; for fulvestrant plus CDK 4/6i was 16.1 months.

### Hormone receptor status

All 150 patients in total were HR+/HER2-, and their distribution according to the percentage of ER and PR positivity was as follows in **Table 2**.

**Table 2:** Hormone receptor status of study population.

Characteristics	Total n (%)
ER ^strong positive^/PR ^strong positive^	49 (32.7%)
ER ^strong positive^/PR ^moderate positive^	31 (20.7%)
ER ^strong positive^/PR ^low positive^	19 (12.7%)
ER ^strong positive^/PR ^negative^	31 (20.7%)
ER ^moderate positive^/PR ^strong positive^	2 (1.3%)
ER ^moderate positive^/PR ^moderate positive^	4 (2.7%)
ER ^moderate positive^/PR ^low positive^	6 (4%)
ER ^moderate positive^/PR ^negative^	8 (5.3%)

### Response evaluation

Among all patients, 86 (57.3%) obtained partial response, 38 (25.3%) had stable disease and 22 (14.7%) had progression. Response assessment could not be performed in four patients (2.7%) at 3 months.

When the response rates according to use of endocrine partner (AI vs fulvestrant respectively) were examined, partial responses were seen in 45 (60%) vs 41 (54.7)%), stable disease in 18 (24%) vs 20 (26.7%), and progression in 9 (12%) vs 13 (17.3%), respectively.

### Toxicity

Grade 3 toxicity was observed in 57 (38%) patients with the most common adverse effect being neutropenia. While no dose reduction was required for ET in the whole group, CDK 4/6i dose reduction was performed in 17 (53.1%) patients in the group receiving AI plus CDK 4/6i and in 15 (46.9%) patients in the group receiving fulvestrant plus CDK 4/6i. Treatment was discontinued in 2 (2.8%) patients in the group receiving AI plus CDK 4/6i due to side effects.

### Survival analysis

**Figure 1:**
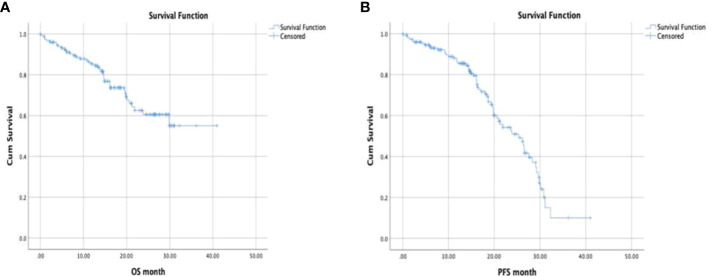
**(A, B)** Progression free survival and overall survival (Kaplan Meier Test).

Median follow-up was 15.2 months (95% CI, 2.1-40.9 months). The median PFS obtained with ET plus CDK 4/6i among all patients was 23.4 months (95% CI, 21.2-25.6) and the median OS was 29.4 months (95% CI, 26.3-32.3) (**Figures 1A, B**).

When outcomes were examined based on endocrine partner (AI vs fulvestrant, respectively) median PFS was 24.4 months (95% CI, 21.8-26.9) vs 22.5 months (95% CI, 20.2-25.8) and medial OS was 25.4 months (95% CI, 22.7-28.2) vs 28.8 (95% CI, 25.0-32.6).

### Effect of hormone receptor positivity percentage on treatment response

The highest response rates were seen in the group with ER^-strong positive^/PR ^moderate or strong positive^, ER ^moderate positive^/PR ^moderate or strong positive^ group, ER ^strong positive^/PR ^negative^ group, and then ER ^moderate positive^/PR ^negative^ group followed in descending order. Although these advantages were not statistically significant, they were numerically higher (ORR: 83.8% vs. 83.3% vs. 77.4% vs. 62.5% p=0.488, respectively). Responses were similar for the first three subgroups, but significantly lower for the ERmoderate positive/PRnegative group (**Table 3**).

**Table 3:** The relationship between hormone positivity and treatment response.

Characteristics	Response Rate	P value
ER^-strong positive^/PR ^moderate or strong positive^,	83.8%	P= 0.488
ER ^moderate positive^/PR ^moderate or strong positive^	83.3%	
ER ^strong positive^/PR ^negative^	77.4%	
ER ^moderate positive^/PR ^negative^	62.5%	

### Effect of hormone receptor positivity percentage on survival

**Figure 2:**
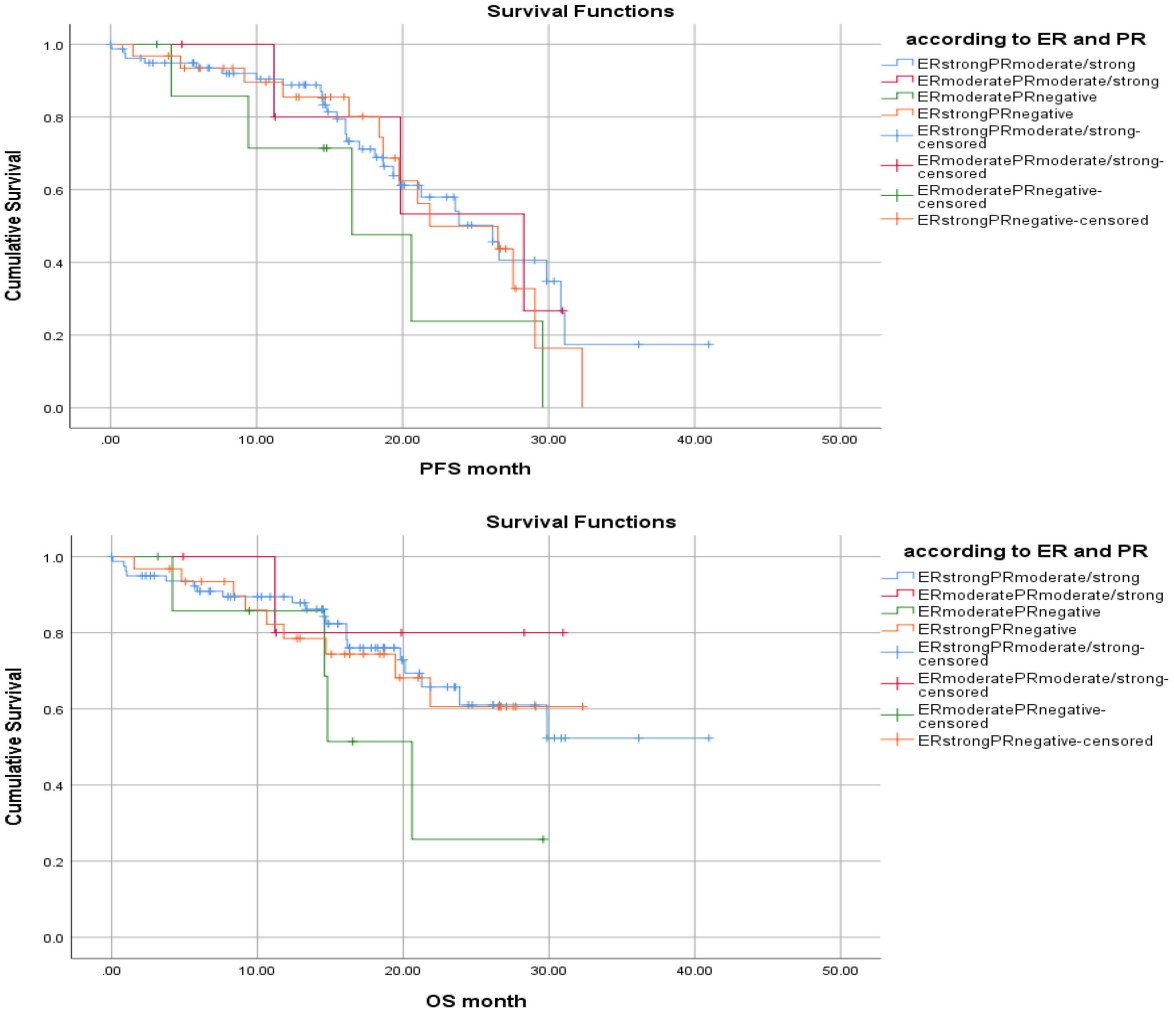
According to status of HR, PFS and OS (Kaplan Meier Test, Log-Rank Analysis).

When the effect of ER and PR positivity percentage on survival in the whole group was analyzed, it was observed that the highest survival was obtained in the ER-^strong positive^/PR ^moderate or strongly positive^ group before treatment, followed by the ER ^moderate positive/^PR ^moderate^ or ^strongly positive^ group, the ER ^strongly positive^/PR ^negative^ group and followed by ER^moderate positive^/PR^negative^ group (mPFS 24.5 vs 23.3 vs 22.6 months vs 17.8 months, p=0.469; mOS 29.6 vs 27.0 months vs 24.7 months vs 18.5 months, p=0.410, respectively). However, these advantages were not statistically significant. Overall survival was similar for the first three subgroups, but significantly shorter for the ER ^moderate-positive/^PR^negative^ group (**Figure 2**). The survival advantage observed in the ER ^strongly positive^/PR ^moderately or strongly positive^ group was similar in both the AI plus CDK 4/6i group and the fulvestrant plus CDK 4/6i group.

Clinicopathological and therapeutic features that may have an impact on survival, and the role of ER and PR positivity percentage were evaluated in univariate and multivariate analysis. The effects of ECOG Performance status, type of endocrine therapy, lung metastases, liver metastases, presence of bone metastases, and percentage of ER and PR positivity on survival in both univariate and multivariate analysis were evaluated. However, no statistical significance was found.

## Discussion

In this study, it was determined that the percentage of baseline HR positivity affected the prognosis in patients with HR+ HER2- MBC treated with CDK 4/6i in combination with an ET. It was observed that the group with ER strong positive/PR moderate or strong positive before treatment was the best prognostic group, followed by the ER moderate positive/PR moderate or strong positive group, followed by the ER moderate positive/PR negative group with a decreasing rate.

One of the most basic features of cancer is the loss of control in cell cycle regulation (28). Normally, the transition from G1 to S phase in the cell cycle is controlled by the Rb gene, through the sequestration of the E2F family transcriptional factor. CDK 4/6, on the other hand, inactivates the Rb gene by forming a complex with D-type cyclins, thus inducing the transition from G1 to S phase (11). Since Cyclin D1 is a direct transcriptional target of estrogen, it has been reported that ER+ tumor cells are particularly dependent on CDK 4/6 activation in cell proliferation (29). In addition, it has been reported that Cyclin D1 amplification is widely observed (29 to 58%) in ER+ breast cancer (30). All these features have paved the way for clinical trials with CDK 4/6i in patients with HR+ HER2- breast cancer. In this sense, studies were conducted in which palbociclib, ribociclib and abemaciclib were combined with ET (12–14). It is observed that these studies were primarily carried out in postmenopausal patients with HR+ HER2- MBC, followed by premenopausal patients, and also studies on its use in the adjuvant setting. In studies in the postmenopausal group, AI or Fulvestrant was used as ET in both metastatic and adjuvant periods; In studies in the premenopausal group, it is seen that tamoxifen or AI is used together with the LHRH analogue. In all these studies, the addition of CDK 4/6i to ET has been shown to contribute significantly to survival and has become one of the main treatment models for MBC.

To date studies of CDK4/6i with ET have been conducted in postmenopausal and premenopausal patients, both in the metastatic and adjuvant settings. In postmenopausal women, CDK4/6i is generally combined with AI or fulvestrant while in premenopausal women, CDK4/6i can be partnered with tamoxifen or AI with LHRH analogue. Across most studies CKD4/6i combined with ET has been shown to improve outcomes. Most Phase III studies with CDK4/6i have included patients with ER+ ≥ 10% and PR+/- (12–14). As a result, the benefit of adding CDK 4/6i to ET was demonstrated in the whole group, and subgroup analysis was also performed according to PR positivity versus negativity. And although there are differences in both analysis and results between studies, the ER+/PR+ group appears to benefit more than the ER+/PR- group. And also in the Paloma-2 study, patients were not stratified by PR positivity however they were in the Monaleesa-2 study (31, 32). In the latter study, a smaller benefit was seen in the PR+ patients when compared to the PR- patients, though the small PR- sample size may have confounded these results. On the other hand, in the Monarch-3 study no significant difference was observed in terms of PR status (33).

We also reviewed the impact of PR positivity or negative in second lines studies of CDK4/6i paired with fulvestrant. In the Paloma-3 study, the role of PR +/- was not found to impart a significant difference.

And neither the efficacy of fulvestrant plus palbociclib nor the likelihood of disease progression more than 6 months after study entry were significantly associated with the level of expression of estrogen or progesterone receptors (HR: 0.32 vs 0.54, respectively) (34). And also in the Paloma-3 study, it was also stated that endocrine sensitivity was a prognostic factor in favor of CDK 4/6i in patients (35). In the Monaleesa-3 and Monarch-2 studies, no significant difference was found between the ER+/PR+ group and the other group (including PR- patients), as well as between the PR+ group and the PR- group, respectively (36, 37). In the Monaleesa-7 study in premenopausal patients receiving CDK4/6i with ET and ovarian function suppression (OFS) no difference was observed between the ER+/PR+ group and the other group (38). In a meta-analysis that included of all phase III studies on CDK 4/6i in metastatic disease, no significant difference was found between the groups in terms of PR status (39). On the other hand, in adjuvant studies of CDK4/6i, such as the MONARCH-E study, benefit was found independent of PR status, although more benefit was obtained in the PR+ group than the PR- group (HR 0.73 vs 0.81) (40).

Many studies in the literature suggest that patients with ER+/PR- tumors have a worse prognosis and higher risk of recurrence than ER+/PR+ tumors (41). In Gharib KE, et al. (42), predictive and prognostic factors were investigated in patients with MBC receiving palbocicilib and letrozole. They reported negative prognostic and predictive features including liver metastases, line of treatment, and absence of PR. The median PFS in PR+ vs PR- groups was 20.05 months vs 12.99 months (p= 0.046) (42).

Similarly, Canino F, et al. (43) evaluated the prognostic role of the intrinsic subtype detected by PAM50 in patients with HR+/HER2- MBC. As a result, it has been reported that the response to ET is low and the prognosis is worse in non-luminal subtypes. In addition, they stated that the response to endocrine therapy was significantly lower in patients whose non-luminal subtype was detected not from the primary tumor but from the metastatic area (43). In a biomarker study conducted in the intrinsic subtypes of the Monaleesa studies, it was stated that the addition of ribociclib to endocrine therapy contributed significantly in all subgroups except the basal-like group. Compared to the luminal A group, the risk of disease progression was found to be 1.44, 2.31 and 3.96 times higher in the Luminal B, HER2-enriched and basal-like groups, respectively (44). Although no separate analysis was performed in the Luminal B group according to PR percentage or Ki-67 index in this analysis, the high risk of progression in this group compared to Luminal A may be an indirect indicator of the importance of hormone receptor positivity.

It has also been postulated that the level of ER expression has a prognostic role in patients with breast cancer undergoing ET (19–22). In Yoon KH et al. patients with ERlow (ER 1-9%) benefitted less fromendocrine therapy and had a significantly higher risk of recurrence compared to the ERhigh group (20). Apart from this, the percentage of hormone receptor positivity as well as its presence or absence have prognostic importance, and in a study conducted by Bae et al., single HR+ tumors without HER2 overexpression (ER + PR-HER2- or ER-PR + HER2-) were found to be of prognostic importance. has been shown to have a poorer survival rate than triple-positive tumors, and this group even has a poorer prognosis comparable to triple-negative breast cancer (45). On the other hand, in the EBCTCG meta-analysis, it was observed that ER positivity was categorized and the benefit from endocrine therapy was highest especially in the group with ER+>50% (18). Similarly, in the P024 study, in which Letrozole and TMX were compared in neoadjuvant therapy in patients with ER+/PR+ breast cancer, patients with ER≥10% positive were included and a linear correlation was reported between ER expression levels and response (46). However, studies investigating the role of hormone receptor levels in the effectiveness of CDK 4/6i are limited in the literature.

Of these, Shikanaj A et al. (47), clinicopathological factors associated with efficacy in patients with MBC were analyzed. As a result, it was reported that tumor grade in the primary lesion and initial neutrophil/lymphocyte ratio (NLR) were associated with efficacy, while expression levels of hormone receptors had no significant effect. In this study, patients were divided into “high” and “low” groups according to the proportion of cells staining positive for ER and PR, and the cut-off value was taken as 66% for this distinction. Patients in which both ER and PR were expressed over 66% were termed the “high” group. However, while separate risk groups were defined for ER and PR, risk groups formed by combining the two were not defined (47). In our study, besides defining separate risk groups for ER and PR according to hormone receptor expression levels, combined risk groups were formed by combining these two. Thus, the prognostic risk groups were better defined and it was determined that the group with ER ^strong positive^/PR ^moderate or strong positive^ was the best prognostic group. This was followed by the ER^moderate positive^/PR^moderate or strongly positive^ group, followed by the ER^moderate positive^/PR^negative^ group in a decreasing fashion. In our study, the positive effect on the prognosis, especially of being ER-strong positive, was more remarkable. In this sense, it was observed that both the response and survival were better in the ER-positive group, even if it was PR-negative. Therefore, our results, although not statistically significant, pointed out the prognostic importance of a high ER positivity. In this respect, it can be thought that our work has a different originality. In the above-mentioned Monaleesa-2 study, the fact that the strong positivity of the ER in both groups was not fully known may also have a role in the lower benefit observed in the PR+ group compared to the PR- group.

In line with all these studies, cyclin D1 is a direct transcriptional target of estrogen and therefore it is known that patients with high hormone receptor expression may obtain more benefit from CDK4/6i when combined with ET.

Our study has some limitations, such as its relatively small sample size, reflecting a single center experience, and retrospective design.

In conclusion, the results of this study suggest that the percentage of HR positivity may have a predictive and prognostic role in patients with HR+ HER-2- MBC who received CDK 4/6i with ET. As far as we know, our study is one of the few studies in the literature conducted with CDK 4/6i in breast cancer patients. We believe that the percentage of hormone receptor positivity and especially the strong positive ER should be taken into account in defining the patient group who will benefit more from the treatment in patients treated with ET plus CDK 4/6i. Our results are hypothesis generating and more comprehensive studies may be needed to further elucidate our findings.

## Data availability statement

The original contributions presented in the study are included in the article/supplementary material. Further inquiries can be directed to the corresponding author.

## Ethics statement

The studies involving humans were approved by Non-Invasive Research Ethics Committee of Dokuz Eylul University Faculty of Medicine. The studies were conducted in accordance with the local legislation and institutional requirements. The participants provided their written informed consent to participate in this study.

## Author contributions

MK: Formal analysis, Methodology, Writing – original draft, Writing – review & editing. HSS: Writing – original draft, Writing – review & editing. TY: Supervision, Writing – original draft, Writing – review & editing. IO: Supervision, Writing – original draft, Writing – review & editing.

## References

[1] Siegel RL Miller KD Jemal A. Cancer statistics, 2020. CA Cancer J Clin. (2020) 70:7–30. doi: 10.3322/caac.21590 31912902

[2] Emens LA. Breast cancer immunotherapy: facts and hopes. Clin Cancer Res. (2018) 24:511–20. doi: 10.1158/1078-0432.CCR-16-3001 PMC5796849 28801472

[3] Nagini S. Breast cancer: current molecular therapeutic targets and new players. Anticancer Agents Med Chem. (2017) 17:152–63. doi: 10.2174/1871520616666160502122724 27137076

[4] Clarke R Tyson JJ Dixon JM. Endocrine resistance in breast cancer—an overview and update. Mol Cell Endocrinol. (2015) 418:220–34. doi: 10.1016/j.mce.2015.09.035 PMC4684757 26455641

[5] Deluche E Antoine A Bachelot T Lardy-Cleaud A Dieras V Brain E. Contemporary outcomes of metastatic breast cancer among 22,000 women from the multicentre ESME cohort 2008–2016. Eur J Cancer. (2020) 129:60–70. doi: 10.1016/j.ejca.2020.01.016 32135312

[6] Anderson WF Chatterjee N Ershler WB Brawley OW. Estrogen receptor breast cancer phenotypes in the Surveillance, Epidemiology, and End Results database. Breast Cancer Res Treat. (2002) 76:27–36. doi: 10.1023/A:1020299707510 12408373

[7] Yue W Yager JD Wang J-P Jupe ER Santen RJ. Estrogen receptor-dependent and independent mechanisms of breast cancer carcinogenesis. Ster- oids. (2013) 78:161–70. doi: 10.1016/j.steroids.2012.11.001 23178278

[8] National Comprehensive Cancer Network. NCCN Clinical Practice Guidelines in Oncology: Breast Cancer v3.2020 (2020). Available online at: https://www.nccn.org/professionals/physician_gls/pdf/breast_blocks.pdf (Accessed 20 Apr 2020).

[9] Files JA Ko MG Pruthi S. Managing aromatase inhibitors in breast cancer survivors: not just for oncologists. Mayo Clin Proc. (2010) 85:560–6. doi: 10.4065/mcp.2010.0137 PMC2878260 20511486

[10] Nagaraj G Ma CX. Clinical challenges in the management of hormone receptor-positive, human epidermal growth factor receptor 2-negative metastatic breast cancer: A literature review. Adv Ther. (2021) 38:109–36. doi: 10.1007/s12325-020-01552-2 PMC7854469 33190190

[11] Finn RS Aleshin A Slamon DJ. Targeting the cyclindependent kinases (CDK) 4/6 in estrogen receptorpositive breast cancers. Breast Cancer Res. (2016) 18:17. doi: 10.1186/s13058-015-0661-5 26857361 PMC4746893

[12] US Food and Drug Administration. Palbociclib highlights of prescribing information (2018). Available online at: https://www.accessdata.fda.gov/drugsatfda_docs/label/2018/207103s007lbl.pdf (Accessed 06 Sep 2018).

[13] US Food and Drug Administration. Ribociclib highlights of prescribing information (2018). Available online at: https://www.accessdata.fda.gov/drugsatfda_docs/label/2018/209092s001lbl.pdf (Accessed 30 Jan 2019).

[14] US Food and Drug Administration. Abemaciclib highlights of prescribing information (2018). Available online at: https://www.accessdata.fda.gov/drugsatfda_docs/label/2018/208855s000lbl.pdf (Accessed 06 Sep 2018).

[15] Spring LM Wander SA Andre F Moy B Turner NC Bardia A. Cyclin-dependent kinase 4 and 6 inhibitors for hormone receptor-positive breast cancer: past, present, and future. Lancet. (2020) 395:817–27. doi: 10.1016/S0140-6736(20)30165-3 32145796

[16] Shah M Nunes MR Stearns V. CDK4/6 inhibitors: game changers in the management of hormone receptor–positive advanced breast cancer? Oncol (willist Park. (2018) 32:216–22. PMC6424488 29847850

[17] Allison KH Hammond MEH Dowsett M McKernin SE Carey LA Fitzgibbons PL. Estrogen and progesterone receptor testing in breast cancer: ASCO/CAP guideline update. Journal of clinical oncology : official journal of the American Society of Clinical Oncology. (2020) 38(12):1346–66. doi: 10.1200/JCO.19.02309 31928404

[18] Early Breast Cancer Trialists’ Collaborative Group (EBCTCG). Relevance of breast cancer hormone receptors and other factors to the effi cacy of adjuvant tamoxifen: patient-level meta-analysis of randomised trials. Lancet. (2011) 378(9793):771–84. doi: 10.1016/S0140-6736(11)60993-8 PMC3163848 21802721

[19] Hill DA Barry M Wiggins C Nibbe A Royce M Prossnitz E. Estrogen receptor quantitative measures and breast cancer survival. Breast Cancer Res Treat. (2017) 166:855–64. doi: 10.1007/s10549-017-4439-6 PMC5681416 28825224

[20] Yoon KH Park Y Kang E Kim EK Kim JH Kim SH. Effect of estrogen receptor expression level and hormonal therapy on prognosis of early breast cancer. Cancer Res Treat. (2022) 54:1081–90. doi: 10.4143/crt.2021.890 PMC9582488 34793665

[21] Van den Eynden GG Colpaert CG Vermeulen PB Weyler JJ Goovaerts G van Dam P. Comparative analysis of the biochemical and immunohistochemical determination of hormone receptors in invasive breast carcinoma influence of the tumor-stroma ratio. Pathol Res Pract. (2002) 198:517–24. doi: 10.1078/0344-0338-00295 12389994

[22] Sparano JA Paik S. Development of the 21-gene assay and its application in clinical practice and clinical trials. J Clin Oncol. (2008) 26:721–8. doi: 10.1200/JCO.2007.15.1068 18258979

[23] Bang YJ Van Cutsem E Feyereislova A Chung HC Shen L Sawaki A. Trastuzumab in combination with chemotherapy versus chemotherapy alone for treatment of HER2-positive advanced gastric or gastro-oesophageal junction cancer (ToGA): a phase 3, open-label, randomised controlled trial. Lancet. (2010) 376:687–97. doi: 10.1016/S0140-6736(10)61121 20728210

[24] Hizal M Bilgin B Paksoy N Atcı MM Kahraman S Kılıçkap S. The percentage of ALK-positive cells and the efficacy of first-line alectinib in advanced non-small cell lung cancer: is it a novel factor for stratification? (Turkish Oncology Group Study). J Cancer Res Clin Oncol. (2022) 149(8):4141–8. doi: 10.1007/s00432-022-04252-2 PMC11798217 36048274

[25] Abdel-Rahman O. Validation of the 8th AJCC prognostic staging system for breast cancer in a population-based setting. Breast. (2018). doi: 10.1007/s10549-017-4577-x 29143220

[26] Eisenhauer EA Therasse P Bogaerts J Schwartz LH Sargent D Ford R. New response evaluation criteria in solid tumours: revised RECIST guideline (version 1.1). Eur J Cancer. (2009) 45:228–47. doi: 10.1016/j.ejca.2008.10.026 19097774

[27] Available online at: https://ctep.cancer.gov/protocoldevelopment/electronic_applications/docs/CTCAE_v5_Quick_Reference_8.5x11.pdf (Accessed March 09, 2018).

[28] Hanahan D Weinberg RA. Hallmarks of cancer: the next generation. Cell. (2011) 144:646–74. doi: 10.1016/j.cell.2011.02.013 21376230

[29] Murphy CG Dickler MN. The role of CDK4/6 inhibition in breast cancer. Oncologist. (2015) 20:483–90. doi: 10.1634/theoncologist.2014-0443 PMC4425391 25876993

[30] Cancer Genome Atlas Network. Comprehensive molecular portraits of human breast tumours. Nature. (2012) 490:61–70. doi: 10.1038/nature11412 23000897 PMC3465532

[31] Finn RS Martin M Rugo HS Jones S Im SA Gelmon K. Palbociclib and letrozole in advanced breast cancer. N Engl J Med. (2016) 375:1925–36. doi: 10.1056/NEJMoa1607303 27959613

[32] Hortobagyi GN Stemmer SM Burris HA. Ribociclib as first-line therapy for HR-positive, advanced breast cancer. N Engl J Med. (2016) 375:1738–48. doi: 10.1056/NEJMoa1609709 27717303

[33] Goetz MP Toi M Campone M. MONARCH 3: abemaciclib as initial therapy for advanced breast cancer. J Clin Oncol. (2017) 35:3638–46. doi: 10.1200/JCO.2017.75.6155 28968163

[34] Cristofanilli M Turner NC Bondarenko I. Fulvestrant plus palbociclib versus fulvestrant plus placebo for treatment of hormone-receptor-positive, HER2-negative metastatic breast cancer that progressed on previous endocrine therapy (PALOMA-3): final analysis of the multicentre, dou- ble-blind, phas. Lancet Oncol. (2016) 17:425–39. doi: 10.1016/S1470-2045(15)00613-0 26947331

[35] Rugo HS Cristofanilli M Loibl S. Prognostic factors for overall survival in patients with hormone receptor-positive advanced breast cancer: analyses from PALOMA-3. Oncologist. (2021) 26(8):e1339–46. doi: 10.1002/onco.13833 PMC8342589 34037282

[36] Slamon DJ Neven P Chia S. Phase III randomized study of ribociclib and fulvestrant in hormone receptor-positive, human epidermal growth factor receptor 2-negative advanced breast cancer: MONALEESA-3. J Clin Oncol. (2018) 36:2465–72. doi: 10.1200/JCO.2018.78.9909 29860922

[37] Sledge GW Jr Toi M Neven P. MONARCH 2: abemaciclib in combination with fulvestrant in women with HR+/HER2- advanced breast cancer who had progressed while receiving endocrine therapy. J Clin Oncol. (2017) 35:2875–84. doi: 10.1200/JCO.2017.73.7585 28580882

[38] Im SA Lu YS Bardia A Harbeck N Colleoni M Franke F. Overall survival with ribociclib plus endocrine therapy in breast cancer. N Engl J Med. (2019) 381:307. doi: 10.1056/NEJMoa1903765 31166679

[39] Johnston SRD Harbeck N Hegg R Toi M Martin M Shao ZM. Abemaciclib combined with endocrine therapy for the adjuvant treatment of HR+, HER2-, node-positive, high-risk, early breast cancer (monarchE). J Clin Oncol. (2020) 38:3987–98. doi: 10.1200/JCO.20.02514 PMC7768339 32954927

[40] Rossi V Berchialla P Giannarelli D Nisticò C Ferretti G Gasparro S. Should all patients with HR-positive HER2-negative metastatic breast cancer receive CDK 4/6 inhibitor as first-line based therapy? A network meta-analysis of data from the PALOMA 2, MONALEESA 2, MONALEESA 7, MONARCH 3, FALCON, SWOG and FACT trials. Cancers. (2019) 11:1661. doi: 10.3390/cancers11111661 31717791 PMC6896062

[41] Cancello G Maisonneuve P Rotmensz N Viale G Mastropasqua MG Pruneri G. Progesterone receptor loss identifies Luminal B breast cancer subgroups at higher risk of relapse. Ann Oncol. (2013) 24:661–8. doi: 10.1093/annonc/mds430 23022996

[42] Gharib KE Macaron W Kattan J Salloum MA Farhat F Smith M. Palbociclib and letrozole in hormone-receptor positive advanced breast cancer: Predictive response and prognostic factors. Curr Problems Cancer. (2022) 46:100859. doi: 10.1016/j.currproblcancer.2022.100859 35378469

[43] Canino F Piacentini F Omarini C. Role of intrinsic subtype analysis with PAM50 in hormone receptors positive HER2 negative metastatic breast cancer: A systematic review. Int J Mol Sci. (2022) 23:7079. doi: 10.3390/ijms23137079 35806079 PMC9266387

[44] Prat A Chaudhury A Solovieff N. Correlative biomarker analysis of intrinsic subtypes and efficacy across the MONALEESA phase III studies. J Clin Oncol. (2021) 39:1458–67. doi: 10.1200/JCO.20.02977 PMC8196091 33769862

[45] Bae SY Kim S Lee JH Lee HC Lee SK Kil WH. Poor prognosis of single hormone receptor- positive breast cancer: similar outcome as triple-negative breast cancer. BMC Cancer. (2015) 15:138. doi: 10.1186/s12885-015-1121-4 25880075 PMC4396721

[46] Ellis MJ Miller WR Tao Y Evans DB Chaudri Ross HA Miki Y. Aromatase expression and outcomes in the P024 neoadjuvant endocrine therapy trial. Breast Cancer Res Treat. (2009) 116:371–8. doi: 10.1007/s10549-008-0161-8 PMC2696016 18941892

[47] Shikanai A Horimoto Y Ishizuka Y Uomori T Nakai K Arakawa A. Clinicopathological features related to the efficacy of CDK4/6 inhibitor-based treatments in metastatic breast cancer. Breast Cancer: Basic Clin Res. (2022) 16:1–9. doi: 10.1177/11782234211065148 PMC8738870 35002243

